# The Week in the Courts

**Published:** 1911-01-21

**Authors:** 


### The Week in the Courts.

### ACTION AGAINST THE ROYAL SOCIETY OF MEDICINE.

In the King's Bench Division of the High Court of Justice, an action was brought by Paul Zotique Hebert, L.R.C.P., against the Royal Society of Medicine to recover damages for an alleged libel and to obtain a declaration that he was entitled to be a member of the Society. Plaintiff's case was that when various medical societies were amalgamated some years ago and the Royal Society of Medicine was formed he sent a postcard applying for membership. Subsequently he published a book, and in an advertisement of it he was described as a member of the Society; the Secretary wrote to the publishers stating that there was no member of the Society of the name of Dr. Hebert; plaintiff complained that this statement was harmful to him. In giving judgment Mr. Justice Darling, who sat without a jury, said the statement might have constituted a libel if plaintiff really were a member. But it was for Dr. Hebert, to succeed in his action, to establish that he was a member. His Lordship did not say that plaintiff did not send the postcard, but he had not proved that he had sent it, and in the absence of such proof there must be judgment for the defendants.

### NEURASTHENIA AND THE WORKMEN'S COMPENSATION ACT.

An application for an award under the Workmen's Compensation Act was made at Maidstone County Court by a carman who, it was stated, suffered from neurasthenia as the result of an accident. A medical practitioner who examined the applicant said he could discover no signs of shock or anything likely to cause neurasthenia, and his Honour Judge Emden, in dismissing the action, said neurasthenia was a complaint which was very little heard of until the Workmen's Compensation Act came into operation, but which now appeared to be increasing rapidly. The number of workmen suffering from neurasthenia was extraordinary, and he felt that none of these workmen knew what the malady was until the Act was passed.

### SURGEON SUED FOR NEGLIGENCE.

In the King's Bench Division, before Mr Justice Phillimore and a special jury, an action was brought by Mr. G. W. Abbott and his wife to recover damages for negligence in the performance of an operation on the female plaintiff by Mr. Charles Ryall. The plaintiffs' case, as stated by Mr. F. E. Smith, K.C., was that on November 5 of 1908 Mr. Ryall performed an operation on Mrs. Abbott, two medical practitioners and two nurses being present at the operation. A few days afterwards Mrs. Abbott experienced pains which her medical attendant attributed to flatulency, and Mr. Ryall called to see her. The pains continued until the following March. Subsequently she called in another medical practitioner, who administered doses of olive oil, and on October 15 Mrs. Abbott passed a swab, which was quite hard. On being informed of this, Mr. Ryall stated that he left the swab in the body to prevent intestinal leakage. Mr. Smith submitted that no surgeon should have sewn a swab of that size in the body of a patient without leaving a vent. Mr. Russell Howard, F.R.C.S., called by plaintiffs' counsel, said he had never left a swab in a body which could not be removed within twenty-four hours. This was a case in which the surgeon might be justified in leaving a swab, but the medical attendant in charge and the nurses should have been told. The practitioner who administered the anæsthetic said he did not see the swab left in, but was not surprised to learn that it had been, considering the character of the operation. Another practitioner who was present at the operation said he did not know a swab was left in, but he considered that Mr. Ryall had acted quite properly in leaving the swab. Sir Alfred Fripp, called by defendant's counsel, Mr. Hume Williams, K.C., said he could well conceive that the leaving of the swab was the best course to take; if he thought the medical man who was to take charge of the case had not seen the swab left he would have told him. Mr. Ryall, in his evidence, described the operation. On opening the abdomen he found there were adhesions, and there was a part of the bowel in which there was a large tear, and he could not break down the adhesions to bring the bowel out of the abdomen. It was necessary to take instant steps to stop possible leakage, and he did the quickest thing—that was to put a swab in the bowel. After the operation he told Nurse Powell, the head nurse, that there was a swab in the bowel which had to be removed, and asked her to attend to the matter. Mr. W. E. Miles, F.R.C.S., and Mr. J. E. Lane, F.R.C.S., said it was a proper thing to put the swab in the intestine as Mr. Ryall had done. Nurse Powell said that after the operation Mr. Ryall instructed her to see if the gauze drain and the plug in the bowel came away; two days afterwards she saw what she believed to be the swab. A week after the operation Mr. Ryall came down to remove the stitches, and she told him that the swab and gauze drain had come away. Mr. Justice Phillimore, in summing up, said that in such a case as this the burden of proof was upon the plaintiffs, and a verdict in their favour could not be returned unless the jury were satisfied that there had been some want of reasonable care or skill on the part of Mr. Ryall. The questions left to the jury were (1) was the defendant guilty of negligence by reason of his leaving the swab in the system; (2) was he guilty of negligence by reason of (*a*) not informing, (*b*) not sufficiently informing persons in charge of the patient that the swab had been left in the system? The jury answered these questions in the negative, and his Lordship entered judgment for Mr. Charles Ryall, with costs.

